# Atypical Scabies Presenting as Annular Plaques: A Case Report and Review of the Literature

**DOI:** 10.7759/cureus.81785

**Published:** 2025-04-06

**Authors:** Aimane Zaim, Hanane Baybay, Fatima Zahra Mernissi

**Affiliations:** 1 Department of Dermatology, Hassan II University Hospital, Fez, MAR

**Keywords:** annular plaques, atypical scabies, dermoscopy, sarcoptes scabiei, ultraviolet dermoscopy

## Abstract

Scabies is a highly contagious parasitic infestation caused by *Sarcoptes scabiei* var. *hominis*, commonly presenting with pruritic lesions such as papules and burrows. While typical scabies lesions are well-documented, atypical presentations can complicate diagnosis. This case report describes an unusual manifestation of scabies presenting as annular plaques. A 50-year-old male with no significant medical history presented with a pruritic rash, including well-defined annular plaques on his thighs. Dermoscopic examination revealed the characteristic "deltaplane" sign and a translucent mite body, consistent with scabies. UV dermoscopy further confirmed the diagnosis with the "ball sign," highlighting the mite's fluorescence. The patient received two doses of oral ivermectin, leading to the complete resolution of pruritus and lesions within 10 days. The rapid resolution and clinical findings led to the diagnosis of atypical scabies. This case highlights the importance of considering atypical scabies in the differential diagnosis of annular plaques and underscores the value of dermoscopy, particularly UV dermoscopy, in enhancing diagnostic accuracy and treatment monitoring in scabies.

## Introduction

Human scabies, caused by the mite *Sarcoptes scabiei* var. *hominis*, is a widespread skin infestation that remains a significant public health concern globally [1]. Clinically, scabies typically presents with pruritic lesions, including papules, vesicles, and nodules, often concentrated in specific body areas. Rarely, it may also present with crusted, pustular, or patchy lesions [2]. The detection of the mite is crucial for diagnosis, and dermoscopy has proven invaluable in enhancing diagnostic accuracy, particularly in atypical cases [3].

This report describes a rare manifestation of scabies presenting as annular plaques, occurring concurrently with typical signs of scabies and with a favorable clinical course. We also explore potential differential diagnoses for annular lesions in scabies, emphasizing the pivotal role of dermoscopy in such cases.

## Case presentation

We report the case of a 50-year-old male with no significant medical history and a good socio-economic background. He sought medical attention for a pruritic rash that developed one week after returning from a trip. The patient reported a worsening of pruritus at night and noted similar symptoms in his wife and children, suggesting potential family transmission.

**Figure 1:**
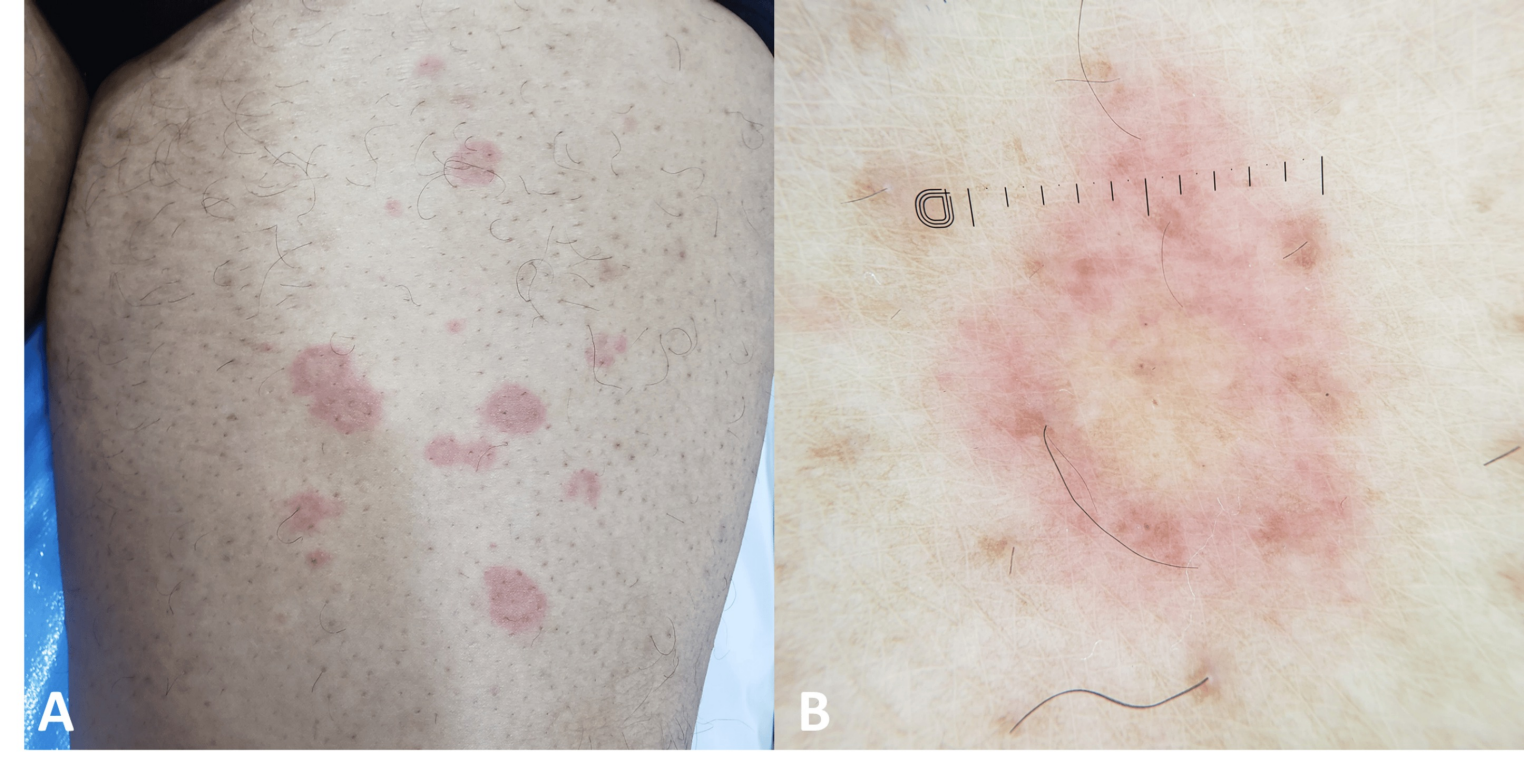
(A) Annular and arciform plaques on the thigh featuring a yellowish center and a well-demarcated erythematous border. (B) Dermoscopy image of the annular lesion showing a structureless, brownish-yellowish central area, encircled by a faint erythematous halo, with no vascular structures, creating a "setting sun" pattern.

**Figure 2:**
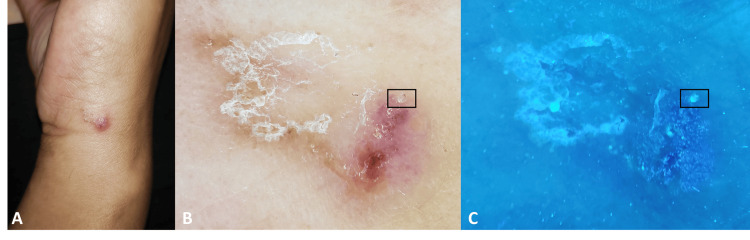
(A) A solitary erythematous-squamous papule with a serpiginous scabies burrow located on the lateral side of the wrist. (B) Dermoscopic image showing a scabies burrow with the characteristic "delta glider" sign, along with the translucent mite body (black square). (C) UV dermoscopic image revealing the green fluorescence of the mite body, known as the "ball sign" (black square).

Dermatological examination revealed multiple annular and arciform plaques on both thighs, each characterized by a yellowish center and a well-defined erythematous border (Figure 1A). Further examination identified several excoriations, a solitary erythematous-squamous papule, and a scabies burrow on the lateral aspect of the wrist (Figure 2A). Dermoscopy of the papule confirmed the presence of a scabies burrow, displaying the characteristic "deltaplane" sign along with the translucent mite body (Figure 2B). UV dermoscopy further supported the diagnosis, showing the pathognomonic "ball sign" (Figure 2C). In contrast, dermoscopy of the annular lesions revealed no significant abnormalities apart from a brownish-yellowish structureless central area, surrounded by a vague erythematous halo, giving a setting sun-like pattern (Figure 1B).

**Figure 3:**
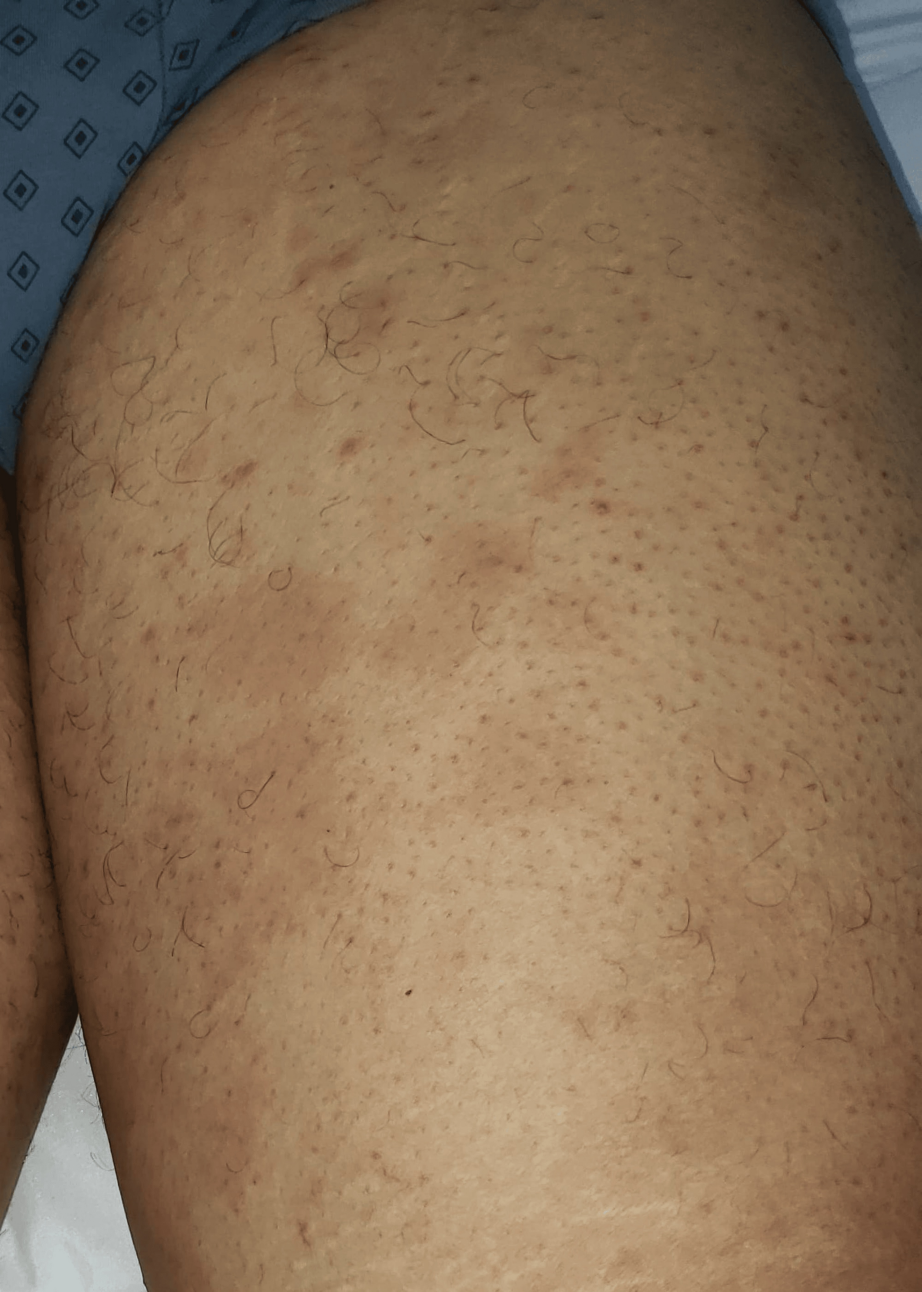
Post-inflammatory hyperpigmentation at the sites of the previous annular lesions on the thigh observed during the 10-day follow-up.

Based on these findings, the diagnosis of annular scabies was established. The patient was treated with two oral doses of ivermectin (200 µg/kg), one week apart, with concurrent treatment for his family. At the 10-day follow-up, the patient reported complete resolution of pruritus, with all lesions having disappeared, leaving only post-inflammatory hyperpigmentation at the sites of the former annular lesions (Figure 3).

## Discussion

Scabies, caused by *Sarcoptes scabiei* var. *hominis*, is a highly contagious parasitic infestation of the skin, typically characterized by an intensely pruritic eruption [1]. Commonly, the lesions appear as papules, vesicles, or burrows, but scabies can sometimes mimic other dermatological conditions, earning it the moniker of the "great imitator" [2].

Accurate diagnosis relies on the detection of the mite, its fecal pellets, or eggs via skin scraping. Dermoscopy has significantly improved the diagnostic accuracy of scabies, with a reported sensitivity of 98.3% and specificity of 88.5% [3]. In polarized dermoscopy mode, several distinctive patterns can be observed, including the "delta glider" sign, which represents the head and anterior legs of the mite; the "jetliner with contrail" sign, where the mite body appears translucent and the air-filled burrow mimics a jetliner with a contrail; and the "gray-edged line" sign, which results from melanin-containing fecal material deposited on the outer wall of the burrow [4,5]. Recently, the "wake sign" has been identified, characterized by wake-shaped scales that resemble the trail left by a moving object on water [5]. Additionally, the "noodle pattern" or "millipede-like structure", formed by multiple curvilinear burrows, is a characteristic dermoscopic feature of crusted scabies [5]. UV dermoscopy has been shown to further enhance diagnostic accuracy, providing clearer and more effective images compared to conventional dermoscopy [6]. In UV mode, the entire mite body emits a bright green fluorescence, known as the "ball sign", while serpentine bright blue burrows with more distinctly defined borders are easily visible, offering superior clarity compared to polarized dermoscopy [7]. Furthermore, UV dermoscopy enhances the visualization of tunnel content, which reflects brightly under UV light [6]. However, the precise chromophore responsible for the observed green fluorescence remains unidentified and warrants further investigation [8]. These features are valuable not only for distinguishing scabies from other pruritic conditions but also for monitoring treatment response.

Annular lesions are seen in a variety of dermatological conditions [9], making their accurate diagnosis challenging without a comprehensive evaluation of the patient's medical history, clinical presentation, and dermoscopic findings. Common conditions that present with annular lesions include erythema migrans, erythema multiforme, lichen planus, pityriasis rosea, plaque psoriasis, tinea corporis, urticaria, and nummular eczema. Less frequently, annular lesions may be indicative of conditions such as fixed drug eruption, granuloma annulare, leprosy, subacute cutaneous lupus erythematosus, immunoglobulin A vasculitis, sarcoidosis, and secondary syphilis [9].

One common condition to consider is erythema multiforme (EM), a hypersensitivity reaction often associated with herpes simplex virus, *Mycoplasma pneumoniae* infections, medications, and vaccinations. EM typically presents with multiple raised, annular, target-like lesions characterized by central erythema and clearing. These lesions develop over a few days and usually resolve within three to five weeks after treatment of the underlying cause [9]. To our knowledge, no cases of EM following scabies infestation have been reported. In our patient, the absence of target-like lesions, the lack of other etiological factors for EM, and the rapid resolution of the lesions within 10 days strongly argue against this diagnosis.

Eczematization, a common complication of scabies [1], can present with nummular eczema, which may overlap with annular lesions. However, the absence of scaling, the lack of the typical patchy distribution of dotted vessels on dermoscopy, and the favorable outcome without steroid treatment make this diagnosis unlikely in our case.

Urticaria, while sometimes associated with scabies [10], is less likely in this case due to the persistent nature of the annular lesions, which lasted longer than 24 hours.

Granuloma annulare (GA), a benign granulomatous condition of uncertain etiology, is another potential differential diagnosis. GA typically presents as firm, shiny papules that may be violaceous, erythematous, or flesh-colored, with central involution leading to non-scaly, annular plaques. These lesions most often occur on the extremities and usually resolve spontaneously within two years [9]. There have been a few case reports linking GA with scabies infection [10-12]. In these cases, scabies infestation was followed by generalized or localized GA. Some researchers have suggested a Koebner phenomenon [11], while others have linked the association to immune dysregulation induced by elevated immunoglobulin E levels in scabies [12]. In most cases, GA lesions appear after at least a month of scabies infestation or treatment, with one exception where GA developed concomitantly with pruritus [11]. Furthermore, the dermoscopic absence of the nearly constant vessel patterns of GA (e.g., unfocused vessels with variable morphology) [13] and the swift resolution of lesions without corticosteroids further argue against GA.

Given the clinical and dermoscopic findings, the diagnosis of atypical scabies presenting with annular plaques is the most reasonable conclusion in our patient’s case. This presentation is exceedingly rare, with only one previous case reporting annular scabies in a 3-year-old girl, confirmed histologically by the identification of a mite egg in the epidermis [14].

## Conclusions

This case underscores the rare possibility of scabies presenting as annular plaques, a phenomenon that could easily lead to misdiagnosis if not properly considered. Therefore, atypical scabies should be included in the differential diagnosis of any pruritic, well-defined, annular lesions. Moreover, dermoscopy, especially UV dermoscopy, offers promising capabilities, significantly enhancing both the accuracy of diagnosis and the ability to monitor treatment response in cases of scabies.
